# On-The-Fly Ambiguity Resolution Based on Double-Differential Square Observation

**DOI:** 10.3390/s18082495

**Published:** 2018-08-01

**Authors:** Tengfei Wang, Zheng Yao, Mingquan Lu

**Affiliations:** Department of Electronic Engineering, Tsinghua University, Beijing 100084, China; wtf13@mails.tsinghua.edu.cn (T.W.); lumq@tsinghua.edu.cn (M.L.)

**Keywords:** ambiguity resolution, ground based positioning, carrier phase measurement

## Abstract

Global navigation systems provide worldwide positioning, navigation and navigation services. However, in some challenging environments, especially when the satellite is blocked, the performance of GNSS is seriously degraded or even unavailable. Ground based positioning systems, including pseudolites and Locata, have shown their potentials in centimeter-level positioning accuracy using carrier phase measurements. Ambiguity resolution (AR) is a key issue for such high precision positioning. Current methods for the ground based systems need code measurements for initialization and/or approximating linearization. If the code measurements show relatively large errors, current methods might suffer from convergence difficulties in ground based positioning. In this paper, the concept of double-differential square observation (DDS) is proposed, and an on-the-fly ambiguity resolution (OTF-AR) method is developed for ground based navigation systems using two-way measurements. An important advantage of the proposed method is that only the carrier phase measurements are used, and code measurements are not necessary. The clock error is canceled out by two-way measurements between the rover and the base stations. The squared observations are then differenced between different rover positions and different base stations, and a linear model is then obtained. The floating integer values are easy to compute via this model, and there is no need to do approximate linearization. In this procedure, the rover’s approximate coordinates are also directly obtained from the carrier measurements, therefore code measurements are not necessary. As an OTF-AR method, the proposed method relies on geometric changes caused by the rover’s motion. As shown by the simulations, the geometric diversity of observations is the key factor for the AR success rate. Moreover, the fine floating solutions given by our method also have a fairly good accuracy, which is valuable when fixed solutions are not reliable. A real experiment is conducted to validate the proposed method. The results show that the fixed solution could achieve centimeter-level accuracy.

## 1. Introduction

Global navigation satellite systems (GNSSs) can provide global navigation and positioning services and are widely used in various industries. As the demand for navigation and positioning grows, GNSS is often combined with other navigation technologies [1,2,3]. In many harsh environments, the reliability or accuracy of GNSS is not satisfactory, such as in the urban canyons and indoor environments, and there have been some solutions [4,5,6,7].

As an alternative solution, ground based positioning systems, including pseudolites (PL) and Locata, are able to provide flexible navigation capabilities to improve the performance of GNSS services. In GNSS denied environments, such systems are able to provide stand-alone services.

The base station includes a transmitter broadcasting GNSS-like signals, from which the receiver obtains code and carrier phase measurements. Ambiguity resolution (AR) is a key issue for high-precision positioning using carrier phase measurements. The AR problem is usually considered as a nonlinear integer-mixed problem. In GNSS high-precision positioning, the geometric changes caused by the motion of satellites and/or receivers help to decorrelate and resolve the ambiguities [8]. In the past decades, the ambiguity resolution (AR) problem has been widely studied for GNSS applications [9,10,11,12,13].

A number of AR methods are proposed for ground based positioning systems. The known-point- initialization (KPI) method accurately measures the initial coordinates, and then calculates the integer ambiguities based on the initial measurements [14]. Their improved particle swarm optimization method requires an initial approximate coordinate with a high precision [15].

However, to do such accurate measurements in advance could be inconvenient for users, and, in practical kinematic applications, in case of loss of signal lock or cycle slips, the receiver has to go back to the initial point and to restart AR. Therefore, the on-the-fly AR (OTF-AR) methods, which resolve integer ambiguities via the geometric diversity, are desirable.

There have been some OTF-AR methods developed for ground based positioning systems. Based on the nonlinear batch least-square estimation, an OTF-AR method is proposed in [16,17]. For GNSS/PL/Inertial Navigation System (INS) integration applications, OTF-AR methods based on the extended Kalman filter are used in [18,19].

In general, existing OTF-AR methods for ground based positioning usually use the code measurements to estimate the approximate coordinates, and then linearize the observation model around these coordinates. Code measurements also provide initial states for the methods that are based on Kalman filters. In GNSS applications, since GNSS satellites are far away from both the receiver and the reference station, the linearization is quite reasonable.

However, in ground based positioning, since the base stations are located nearby the receiver, such linearization might cause significant nonlinear errors [20]. This might bring some difficulties for the OTF-AR methods above. Compared with the carrier measurements, the thermal noise of code measurements is much larger and the multipath effect is also more severe. Moreover, in ground positioning applications, since the base stations are close to the receiver, the errors caused by linearization cannot be directly ignored [20]. As a result, the AR success rate greatly depends on the accuracy of code or other measurements made in advance, and a poor initial estimate might lead to convergence difficulties [19].

In some cases, such as the indoor environment, the base stations and the receiver are very close. The code measurements might suffer from errors comparable with the true distance, and thus fail to provide sufficiently accurate coordinates. In this situation, the AR success rates of current methods could be quite poor.

In this paper, a new OTF-AR method based on double-differential square (DDS) observations is proposed. This method is suitable for ground based positioning systems using two-way measurements. The two-way ranging is able to eliminate the clock errors at the cost that the user equipment includes a transmitter.

Two-way ranging has been widely used in wireless positioning [21,22,23], and related high-precision applications using carrier phase measurements can be found in [24,25]. Such a system usually consists of several stationary base stations for which coordinates are known, and a kinematic rover that can receive and transmit ranging signals.

Similar to existing OTF-AR methods, the rover’s motion is necessary to cause geometric changes since the base stations are stationary. The rover collects carrier phase measurements at different sampling points during its movements. These measurements are squared and differenced twice to obtain a linear model. It is shown by the analysis that, with a sufficient number of sampling points, the model is able to be solved, without other measurements made in advance.

An important feature of the proposed method is the concept of the DDS observation. The measurements at different points are squared first. The squared observations are then differenced between different points and base stations, and, in this way, a linear model is obtained. The proposed method provides a new way to utilize the geometric changes for resolving ambiguities.

The proposed method is able to directly obtain approximate coordinates and floating integer values from this model, and this is the reason why other measurements are not necessary. To the best of the author’s knowledge, there is no previous method that solely relies on carrier phase measurements. The proposed method is free from the influences of other measurements, which could be an important advantage. In other words, it applies to cases where other measurements are not accurate enough or may be unavailable.

The remainder of this paper is organized as follows. In Section 2, we give a brief review of the two-way measurement model. In Section 3, the concept of DDS observation is introduced to establish a linear model, and the procedure of resolving ambiguities is specified based on the noise analysis. In Section 4, several simulations show the validity of the proposed method. The influence of the number of sampling points, and the number of base stations are discussed. It is shown that the geometric diversity actually has an important influence on the AR success rate. A real experiment is conducted to validate the proposed method, and the results are shown and discussed in Section 5. Finally, conclusions are drawn in Section 6.

## 2. Problem Formulation

We consider a positioning problem for a ground based positioning system using two-way ranging [24,25]. Suppose that there are *L* base stations denoted as ${\mathrm{BS}}_{1},\;{\mathrm{BS}}_{2},...,\;{\mathrm{BS}}_{L}$. The coordinates of ${\mathrm{BS}}_{i}$ are precisely measured and denoted as $s_{i}={\left(x_{i}^{s},\;y_{i}^{s},\;z_{i}^{s}\right)}^{T}$. The rover’s coordinate and carrier phase measurement to ${\mathrm{BS}}_{i}$ at *k*-th position are denoted as $u_{k}={\left(x_{k}^{u},\;y_{k}^{u},\;z_{k}^{u}\right)}^{T}$ and $\phi_{k}^{i}$, respectively. The measurement $\phi_{k}^{i}$ depends on ${\mathrm{BS}}_{i}$’s coordinates $s_{i}$, the rover’s coordinate $u_{k}$, ${\mathrm{BS}}_{i}$’s clock error $\delta t_{i}$, and the rover’s clock error $\delta t_{k}$ as follows:

(1) $$\phi_{k}^{i}=\frac{1}{\lambda }∥s_{i}-u_{k}∥+N_{i}+\frac{c}{\lambda }\left(\delta t_{i}-\delta t_{k}\right)+w_{k}^{i},$$

where ∥·∥ represents the Euclidean distance, λ is the carrier wavelength, $N_{i}$ is the unknown integer value, *c* is the speed of light, and $w_{k}^{i}$ is the observation noise. Since the multipath error varies at different positions, in this paper, it is considered as a kind of noise instead of a series of constant biases. The maximum multipath error is no larger than λ/4, and there are ways to mitigate the multipath effects for ground based positioning via choke ring antenna, spatial, polarization or frequency design techniques [26,27].

For GNSS applications, the double-differential observation can eliminate the effects of clock errors, and a reasonable linearization is used. For ground based positioning using carrier phase measurements, the self-differential two-way ranging technique has been proposed, where both the rover and base stations are able to transmit and receive signals [24,25]. The ${\mathrm{BS}}_{i}$’s measurement to the rover is written as

(2) $${\tilde{\phi }}_{k}^{i}=\frac{1}{\lambda }∥s_{i}-u_{k}∥+{\tilde{N}}_{i}+\frac{c}{\lambda }\left(\delta t_{k}-\delta t_{i}\right)+{\tilde{w}}_{k}^{i}.$$

With the two-way measurements, the clock errors in Equation (1) are canceled, and the observation is written as follows:

(3) $$\theta_{k}^{i}=\frac{2}{\lambda }∥s_{i}-u_{k}∥+Z_{i}+n_{k}^{i}$$

where $\theta_{k}^{i}=\phi_{k}^{i}+{\tilde{\phi }}_{k}^{i}$, $Z_{i}=N_{i}+{\tilde{N}}_{i}$ and $n_{k}^{i}=w_{k}^{i}+{\tilde{w}}_{k}^{i}$. $Z_{i}$ denotes the the new ambiguity for two-way measurements.

Before the carrier phase measurements can actually be used in position determination, the ambiguities $Z_{i}$ should be accurately resolved. The geometric changes is necessary to decorrelate the ambiguities. Since the ground based base stations are stationary, the geometric changes relies on the rover’s motion. During the rover’s motion, the observations described in (3) are collected at different positions. It is assumed that there is no loss of signal lock or cycle slip, so that the carrier phase integers are constant during this procedure.

With a sufficient number of observations, the OTF-AR problem is solvable in theory. In previous methods, a typical solution of similar problems is to perform a type of approximate linear expansion, in which the code measurements are used. As discussed in the introduction, if the code phase measurements are of a relatively low accuracy, methods using such a linear expansion will encounter convergence difficulties and this might be a flaw in some cases.

In the next section, we propose the concept of double-differential square observation, which provides a new way to establish a linear model. It will be seen that only carrier phase observations are involved in solving this linear model, and, at the same time, floating integer values and approximate coordinates are obtained.

## 3. The Proposed Method

### 3.1. Double-Differential Square Observation

As the naming of DDS shows, the observation in Equation (3) are firstly squared as

(4) $${\left(\theta_{k}^{i}-Z_{i}\right)}^{2}=(\frac{2}{\lambda }∥s_{i}-u_{k}{∥+n_{k}^{i})}^{2}.$$

With some manipulations, the square observation ${\left(\theta_{k}^{i}\right)}^{2}$ is obtained as follows:

(5) $${\left(\theta_{k}^{i}\right)}^{2}=\frac{4∥s_{i}-u_{k}{∥}^{2}}{\lambda^{2}}+2\theta_{k}^{i}Z_{i}-Z_{i}^{2}+\frac{4∥s_{i}-u_{k}∥n_{k}^{i}}{\lambda }+{\left(n_{k}^{i}\right)}^{2}.$$

Similar to the traditional writing of GNSS differencing symbols, we define the single-differential square (SDS) observation between position $u_{k}$ and $u_{m}$ as $\Delta^{2}\theta_{km}^{i}={\left(\theta_{k}^{i}\right)}^{2}-{\left(\theta_{m}^{i}\right)}^{2}$. With Equation (5), $\Delta^{2}\theta_{km}^{i}$ is written as

(6) $$\begin{array}{cc} \Delta^{2}\theta_{km}^{i}= & {\left(\theta_{k}^{i}\right)}^{2}-{\left(\theta_{m}^{i}\right)}^{2}\\ = & \frac{4[-2s_{i}^{T}\left(u_{k}-u_{m}\right)+u_{k}^{T}u_{k}-u_{m}^{T}u_{m}]}{\lambda^{2}}+2\left(\theta_{k}^{i}-\theta_{m}^{i}\right)Z_{i}\\ + & \frac{4(∥s_{i}-u_{k}∥n_{k}^{i}-∥s_{i}-u_{m}∥n_{m}^{i})}{\lambda }+{\left(n_{k}^{i}\right)}^{2}-{\left(n_{m}^{i}\right)}^{2}. \end{array}$$

Generally, the levels of measurement errors are considered to be much smaller than the true distances, or it will be impossible to perform carrier phase positioning. In this regard, we have $∥s_{i}-u_{k}∥\gg \lambda n_{k}^{i}$ and $∥s_{i}-u_{m}∥\gg \lambda n_{m}^{i}$, and the equation above can be simplified as follows:

$$\Delta^{2}\theta_{km}^{i}=\frac{4[-2s_{i}^{T}\left(u_{k}-u_{m}\right)+u_{k}^{T}u_{k}-u_{m}^{T}u_{m}]}{\lambda^{2}}+2\left(\theta_{k}^{i}-\theta_{m}^{i}\right)Z_{i}+\frac{4(∥s_{i}-u_{k}∥n_{k}^{i}-∥s_{i}-u_{m}∥n_{m}^{i})}{\lambda }.$$

It can be seen that the quadratic term of the ambiguity $Z_{i}^{2}$ is eliminated by single-differencing in the SDS observation.

There are still quadratic terms of the unknown coordinates in Equation (6), which are $u_{k}^{T}u_{k}$ and $u_{m}^{T}u_{m}$. These two quadratic terms can be eliminated by differencing between $PL_{i}$ and $PL_{j}$, and leads to the double-differential square observation. The definition of DDS observation $\nabla \Delta^{2}\theta_{km}^{ij}$ is as follows:

(7) $$\begin{array}{cc} \nabla \Delta^{2}\theta_{km}^{ij}= & {\left(\theta_{k}^{i}\right)}^{2}-{\left(\theta_{k}^{j}\right)}^{2}-{\left(\theta_{m}^{i}\right)}^{2}+{\left(\theta_{m}^{j}\right)}^{2}\\ = & -\frac{8{\left(s_{i}-s_{j}\right)}^{T}\left(u_{k}-u_{m}\right)}{\lambda^{2}}+2\left(\theta_{k}^{i}-\theta_{m}^{i}\right)Z_{i}-2\left(\theta_{k}^{j}-\theta_{m}^{j}\right)Z_{j}\\ & +\frac{4(∥s_{i}-u_{k}∥n_{k}^{i}-∥s_{i}-u_{m}∥n_{m}^{i})}{\lambda }-\frac{4(∥s_{j}-u_{k}∥n_{k}^{j}+∥s_{j}-u_{k}∥n_{m}^{j})}{\lambda }. \end{array}$$

In the above, the definition of DDS observation is clarified, and, with twice differencing, several quadratic items are eliminated. The unknown items in the last two lines of Equation (7) are considered as noise items, and, in this way, a linear observation model is established.

The rover moves and collects measurements at different positions to obtain DDS observations. For example, the carrier phase measurements collected at $u_{k}$ and $u_{m}$ are converted to L−1 DDS observations as

(8) $$\underset{y_{km}}{\underset{︸}{\left[\begin{array}{c} \nabla \Delta^{2}\theta_{km}^{21}\\ \nabla \Delta^{2}\theta_{km}^{31}\\ \vdots \\ \nabla \Delta^{2}\theta_{km}^{L1} \end{array}\right]}}=\underset{\tilde{S}}{\underset{︸}{\frac{8}{\lambda^{2}}\left[\begin{array}{c} {\left(s_{1}-s_{2}\right)}^{T}\\ {\left(s_{1}-s_{3}\right)}^{T}\\ \vdots \\ {\left(s_{1}-s_{L}\right)}^{T} \end{array}\right]}}u_{km}+\Phi_{km}\underset{z}{\underset{︸}{\left[\begin{array}{c} Z_{1}\\ Z_{2}\\ \vdots \\ Z_{L} \end{array}\right]}}+\underset{n_{km}}{\underset{︸}{\left[\begin{array}{c} n_{km}^{21}\\ n_{km}^{31}\\ \vdots \\ n_{km}^{L1} \end{array}\right]}},$$

where $u_{km}=u_{k}-u_{m}$,

(9) $$\Phi_{km}=2\left[\begin{array}{ccccc} \theta_{m}^{1}-\theta_{k}^{1} & \theta_{k}^{2}-\theta_{m}^{2} & & & \\ \theta_{m}^{1}-\theta_{k}^{1} & & \theta_{k}^{3}-\theta_{m}^{3} & & \\ \theta_{m}^{1}-\theta_{k}^{1} & & & \ddots & \\ \theta_{m}^{1}-\theta_{k}^{1} & & & & \theta_{k}^{L}-\theta_{m}^{L} \end{array}\right]$$

and

$$\begin{array}{cc} n_{km}^{ij}= & \frac{4(∥s_{i}-u_{k}∥n_{k}^{i}-∥s_{i}-u_{m}∥n_{m}^{i})}{\lambda }-\frac{4(∥s_{j}-u_{k}∥n_{k}^{j}+∥s_{j}-u_{m}∥n_{m}^{j})}{\lambda }. \end{array}$$

Then, rewrite the equations in (8) as

(10) $$y_{km}=\tilde{S}u_{km}+\Phi_{km}z+n_{km},$$

where $y\in R^{(L-1)\times 1}$, $\tilde{S}\in R^{(L-1)\times 3}$, $\Phi_{km}\in R^{(L-1)\times L}$ , $z={\left(Z_{1},\;Z_{2},\;...,\;Z_{L}\right)}^{T}$, $n_{km}\in R^{(L-1)\times 1}$.

In Equation (8), the unknown terms are the differential coordinates and integer values. Every time the rover moves to a new position, new L−1 DDS observations are obtained along with a new differential coordinate.

If the rover collects all observations at *K* different positions, the total number of DDS observations is (L−1)(K−1). Then, a linear observation model is obtained by stacking Equation (10) for m=1 and k=2,3,…,K in matrix form

(11) $$\underset{y}{\underset{︸}{\left[\begin{array}{c} y_{21}\\ y_{31}\\ \vdots \\ y_{K1} \end{array}\right]}}=\underset{S}{\underset{︸}{\left[\begin{array}{cccc} \tilde{S} & & & \\ & \tilde{S} & & \\ & & \ddots & \\ & & & \tilde{S} \end{array}\right]}}\underset{u}{\underset{︸}{\left[\begin{array}{c} u_{21}\\ u_{31}\\ \vdots \\ u_{K1} \end{array}\right]}}+\underset{\Phi }{\underset{︸}{\left[\begin{array}{c} \Phi_{21}\\ \Phi_{31}\\ \vdots \\ \Phi_{K1} \end{array}\right]}}z+\underset{n}{\underset{︸}{\left[\begin{array}{c} n_{21}\\ n_{31}\\ \vdots \\ n_{K1} \end{array}\right]}},$$

where $y\in R^{(L-1)(K-1)\times 1}$, $u\in R^{(K-1)\times 3}$ and $z\in Z^{L\times 1}$ represent the vectors of DDS observation, rover’s differential coordinates and integer values, respectively. Matrices S and Φ come from the known coordinates of base stations and carrier phase measurements, respectively.

As shown above, the DSS observation model in (11) is linear, and its solvability condition is able to be directly obtained. For a *D*-dimensional case, the number of the unknown values in (11) is *L* integer values and D(K−1) differential rover’s coordinates (D=3 in this paper if there is no special definition). The solvability condition is (L−1)(K−1)≥L+D(K−1), and it is not difficult to be decomposed into two inequalities:

(12) $$\begin{array}{cc} L & \geq D+2, \end{array}$$

(13) $$\begin{array}{cc} K & \geq \frac{L}{L-D-1}+1. \end{array}$$

The first condition in (12) constrains the minimum number of base stations. To satisfy the second condition (13), enough observations at different positions should be collected to ensure geometric diversities and resolve ambiguities.

### 3.2. The Procedure of Resolving Ambiguities

The solution of model (11) is given by a mixed integer least-squares problem

(14) $$\min_{z\in Z^{L},\;u\in R^{3\times (K-1)}}\;{∥y-Su-\Phi z∥}_{Q_{\mathrm{yy}}}^{2},$$

where ${∥\cdot ∥}_{Q_{\mathrm{yy}}}^{2}={\left(\cdot \right)}^{T}Q_{\mathrm{yy}}^{-1}\left(\cdot \right)$ and $Q_{\mathrm{yy}}=E\left\{nn^{T}\right\}$. This problem is a mixed-integer problem, and its form is similar to those in GNSS positioning.

Generally, in order to use typical integer searching algorithms such as least-squares ambiguity decorrelation adjustment (LAMBDA) method [9,12], it is necessary to know the variance matrix $Q_{\mathrm{yy}}$ as well as an initial solution. Denoting the variance of $n_{k}^{i}$ as $\sigma_{ik}^{2}$, it can be obtained that

(15) $$Q_{\mathrm{yy}}=\left[\begin{array}{cccc} E\{n_{21}n_{21}^{T}\} & E\{n_{21}n_{31}^{T}\} & ... & E\{n_{21}n_{K1}^{T}\}\\ E\{n_{31}n_{21}^{T}\} & E\{n_{31}^{T}n_{31}\} & ... & E\{n_{31}n_{K1}^{T}\}\\ \vdots & \vdots & \ddots & \vdots \\ E\{n_{K1}n_{21}^{T}\} & E\{n_{K1}^{T}n_{31}\} & ... & E\{n_{K1}n_{K1}^{T}\}. \end{array}\right]$$

For k=2,3,…,L, we have

(16) $$\begin{array}{cc} & E\{n_{k1}n_{k1}^{T}\}\\ = & \frac{16}{\lambda^{2}}\left[\begin{array}{ccc} \left|\right|s_{2}-u_{k}{\left|\right|}^{2}\sigma_{2k}^{2} & & \\ & \ddots & \\ & & \left|\right|s_{L}-u_{k}{\left|\right|}^{2}\sigma_{Lk}^{2} \end{array}\right]+\frac{16}{\lambda^{2}}\left[\begin{array}{ccc} \left|\right|s_{2}-u_{1}{\left|\right|}^{2}\sigma_{21}^{2} & & \\ & \ddots & \\ & & \left|\right|s_{L}-u_{1}{\left|\right|}^{2}\sigma_{L1}^{2} \end{array}\right]\\ + & \frac{16}{\lambda^{2}}\left(|\right|s_{1}-u_{k}{\left|\right|}^{2}\sigma_{k}^{2}+\left|\right|s_{1}-u_{1}{\left|\right|}^{2}\sigma_{11}^{2})1_{L-1}1_{L-1}^{T}\}, \end{array}$$

where $1_{L-1}$ is a column vector of L−1 ones. For p=2,3,…,L and p≠k, we have

(17) $$\begin{array}{cc} & E\{n_{k1}^{T}n_{p1}\}\\ = & \frac{16}{\lambda^{2}}\left[\begin{array}{ccc} \left|\right|s_{2}-u_{1}{\left|\right|}^{2}\sigma_{21}^{2} & & \\ & \ddots & \\ & & \left|\right|s_{L}-u_{1}{\left|\right|}^{2}\sigma_{L1}^{2} \end{array}\right]+\frac{16}{\lambda^{2}}\left|\right|s_{1}-u_{k}{\left|\right|}^{2}\sigma_{11}^{2}1_{L-1}1_{L-1}^{T}. \end{array}$$

As we can see in (16) and (17), the expression of $Q_{\mathrm{yy}}$ is related to the rover’s coordinates, which are unknown. Thus, it is a priority issue to obtain $Q_{\mathrm{yy}}$ without the information of the rover’s coordinates. It should be noted that the solution of Equation (14) directly gives the floating integer values and differential coordinates. If there is no reliable knowledge of approximate coordinates, a unit matrix can be used as the weighting matrix in problem (14) instead of $Q_{\mathrm{yy}}$. The integer constraint on z is relaxed, and, in this way, an initial solution consisting of the floating point integer values is quite easy to compute.

In this way, the proposed method is able to get approximate coordinates by carrier phase measurements to estimate $Q_{\mathrm{yy}}$, instead of involving other means. With this initial solution, fine solutions can be obtained and the LAMBDA algorithm is used to search the integer solution. The specific steps of the AR procedure are as follows:

1. Obtain a raw floating point solution $\hat{z}$ by solving the unweighted least-squares problem
  (18) $$\underset{z\in R^{L}}{\hat{z}=\mathrm{argmin}}{∥y-Su-\Phi z∥}^{2}.$$
2. According to the raw floating point solution $\hat{z}$, calculate the rover’s coordinates with two-way measurements in Formula (3). Then, the estimate of variance matrix ${\hat{Q}}_{\mathrm{yy}}$ is obtained via (15), (16) and (17).
3. Refine the solution $\hat{z}$ with the estimate ${\hat{Q}}_{\mathrm{yy}}$ by solving the following weighted least-squares problem
  (19) $$\tilde{z}=\underset{z\in R^{L}}{\mathrm{argmin}}{∥y-Su-\Phi z∥}_{{\hat{Q}}_{\mathrm{yy}}}^{2}.$$
4. Compare the raw floating point solution $\hat{z}$ and fine floating point solution $\tilde{z}$. If $\hat{z}$ and $\tilde{z}$ are the same after rounding, proceed to the next step; otherwise, let $\hat{z}:=\overset{ˇ}{z}$ and return to the second step.
5. Obtain a new estimate of variance matrix ${\tilde{Q}}_{\mathrm{yy}}$ by the fine floating point solution $\tilde{z}$.
6. Input $\overset{ˇ}{z}$ as the initial floating point solution, and search the integer solution $\overset{ˇ}{z}$ by LAMBDA algorithm
  (20) $$\overset{ˇ}{z}=\underset{z\in Z^{L}}{\mathrm{argmin}}{∥y-Su-\Phi z∥}_{{\tilde{Q}}_{\mathrm{yy}}}^{2}.$$

It can be seen that, in the first two steps, the rover’s approximate coordinates are obtained only using the carrier phase measurements. This provides initial information to estimate $Q_{\mathrm{yy}}$ in the following steps. If there are a priori coordinates with sufficient accuracy, the procedure above could be modified, but that is not the focus of this paper.

## 4. Simulation Results

As seen from the previous discussion, the proposed method only relies on the carrier phase measurements, while the performance of other existing methods is influenced by the code phase measurements. As a result, it may be difficult to make meaningful and fair comparisons. Taking this into account, simulations in this paper are mainly focused on the impact of key parameters for the proposed method.

**Figure 1:**
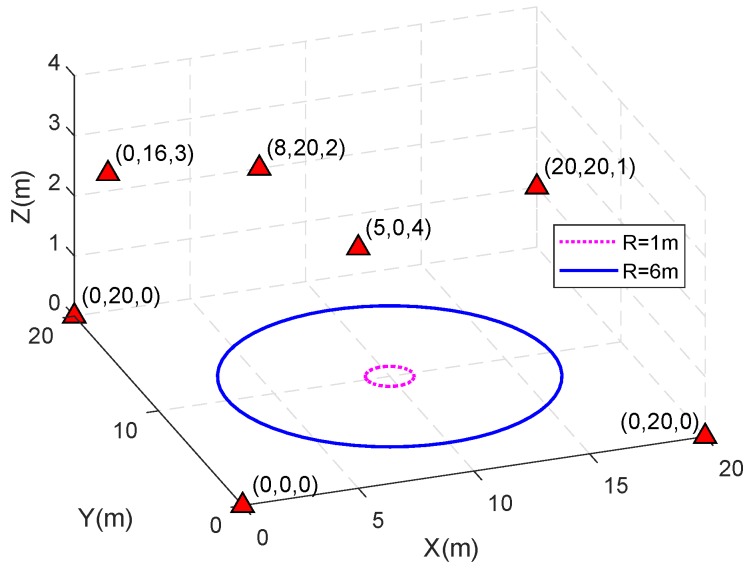
The schematic illustration of the locations of base stations and rover’s movements. The blue larger circle has a radius of 6 m, while the smaller one has a radius of 1 m.

As shown in Figure 1, seven base stations are placed in a 20 m × 20 m × 4 m space, with their positions marked by the red triangle. The carrier frequency of the signal is 1575.42 MHz, therefore the carrier wavelength is about 19 cm. The accuracy of the carrier phase measurements should be carefully set. For GNSS, the accuracy of carrier phase measurements are usually assumed to be about 0.01 cycles [28]. However, in ground based positioning, the multipath effects sometimes are more severe than those in GNSS applications. There have been several solutions discussed [26,27].

In the following, we set two noise levels for the original measurement in (1): the standard deviation of error is set to be σ=0.05 cycle and σ=0.1 cycle, respectively. The noise is assumed to be normally distributed.

The rover moves along a horizontal circle centered at (10,10,0), and the radius is denoted as *R*. To decouple the influences of sampling rate and geometric changes, it is assumed that the rover has a fixed speed, and just moves only once along the trajectory. The observations are collected at *K* positions uniformly spaced around the circle, and a larger *K* means denser sampling points in space and a higher sampling rate.

For a specific *K* and *R*, 100 trials are carried out. In the last step of the AR procedure, the LAMBDA algorithm always outputs the best integer solutions.

### 4.1. Influences of Movement Radius and Sampling Density

**Figure 2:**
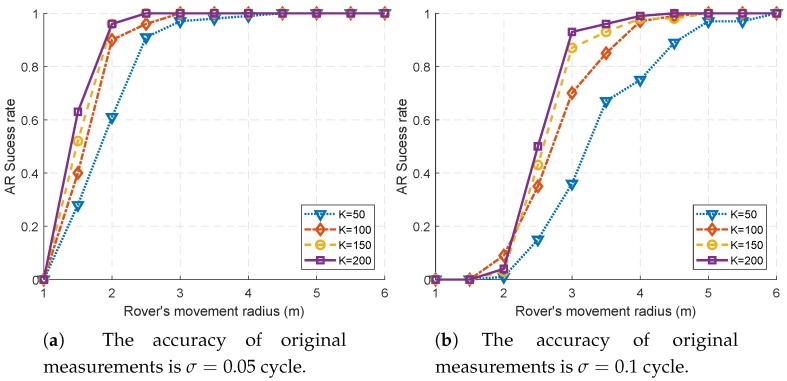
The comparison of AR success rates. The rover’s movement radius is increased from 1 m to 6 m.

First, we study the influence of geometric changes on the AR success rate, which are caused by the rover’s motion. As can be seen in Figure 2a,b, the AR success rate is significantly improved as the rover’s motion radius increases. In fact, since the base stations are stationary, only the rover’s motion contributes to geometric diversity. Such geometric diversity helps to decorrelate the ambiguities rapidly. This result is just in line with the intuition, as well as GNSS applications.

From Figure 2a,b, it can be seen that, for a certain *R*, as the number of sampling points *K* increases, the AR success rate also increases to some extent. Comparing the results of K=50 and K=100, the benefit from increasing the number of samples is significant. However, increasing *K* is not always so effective. Comparing the results of K=150 and K=200 in Figure 2a,b, the margin of benefit to AR success rate declines.

This is because the trajectory is a fixed circle, and a larger *K* means denser points in space. When *K* is relatively small, more sampling points will provide additional geometric diversity, while this effect is not so significant with a large *K*.

Although in the simulations increasing the number of samples can bring more or less benefit, the benefits of overly intensive sampling are questionable in practical applications because the errors caused by multipath may be relevant for sampling points that are very close together.

In general, to resolve ambiguities, expanding a rover’s motion range is the most direct and effective way. By comparing the accuracy of the raw floating point solution and the fixed floating point solution, this issue will be better illustrated in the following.

### 4.2. Comparison of Raw Solutions, Fine Solutions

Furthermore, we compare the raw floating point solution $\hat{z}$ and fine floating point solution $\tilde{z}$, which are given by the AR procedure in the first and fifth step. Their errors are evaluated by

(21) $${\hat{e}}^{2}=\frac{1}{L}\left|\right|\hat{z}-{z||}^{2},\;{\tilde{e}}^{2}=\frac{1}{L}\left|\right|\tilde{z}-z{\left|\right|}^{2}.$$

$\hat{e}$ and $\tilde{e}$ indicate the estimate accuracy in floating ambiguities.

**Figure 3:**
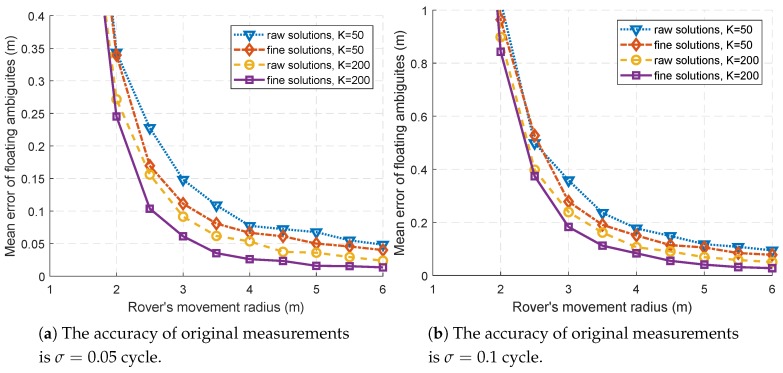
The comparison of mean errors between the raw and fine floating point solutions. The rover’s movement radius *R* is increased from 1 m to 6 m, and the results with R<2 m are rather bad and not conducive to display.

The simulation results with σ=0.05 cycle and σ=0.1 cycle are shown in Figure 3a,b, respectively. When the range of motion is too small (R<2 m), that is, when the geometric diversity is insufficient, the results are too bad to display.

It is shown that, in most cases, the fine floating point solutions have better accuracy than the raw ones with either noise level. The raw floating point solution $\hat{z}$ is of decimeter accuracy when R>2 m as shown in Figure 3. Based on $\hat{z}$, the proposed method estimates $Q_{\mathrm{yy}}$ and then obtains a fine solution $\tilde{z}$ with a better accuracy. This demonstrates the effectiveness of refining the solution in the third step of the proposed AR procedure.

On the other hand, it can be clearly seen that the accuracy of both solutions is improved as the rover’s movement radius *R* increases. When the accuracy of the floating solution is poor, a reliable integer solution is not possible and, as shown in Figure 2a,b, the AR success rate could be very low. It can be said that the benefits of geometric diversity can be reflected in the first four steps of the AR procedure, not just the last step. Furthermore, floating point solutions with higher accuracy will also help to resolve ambiguities successfully.

In practical applications, the motion trajectory is not limited to be a circle and can be more casual, and, in general, the increase in geometric diversity is quite helpful for improving the accuracy of floating point solutions and resolving integer ambiguities.

### 4.3. Comparison of Positioning Accuracy of Float Solutions and Fixed Solutions

Next, we compare positioning accuracy using floating point solutions and fixed solutions. For the sake of comparison, the integer solution output by the LAMDBA algorithm is always used for positioning, even if the AR success rate could be very low under current conditions.

**Figure 4:**
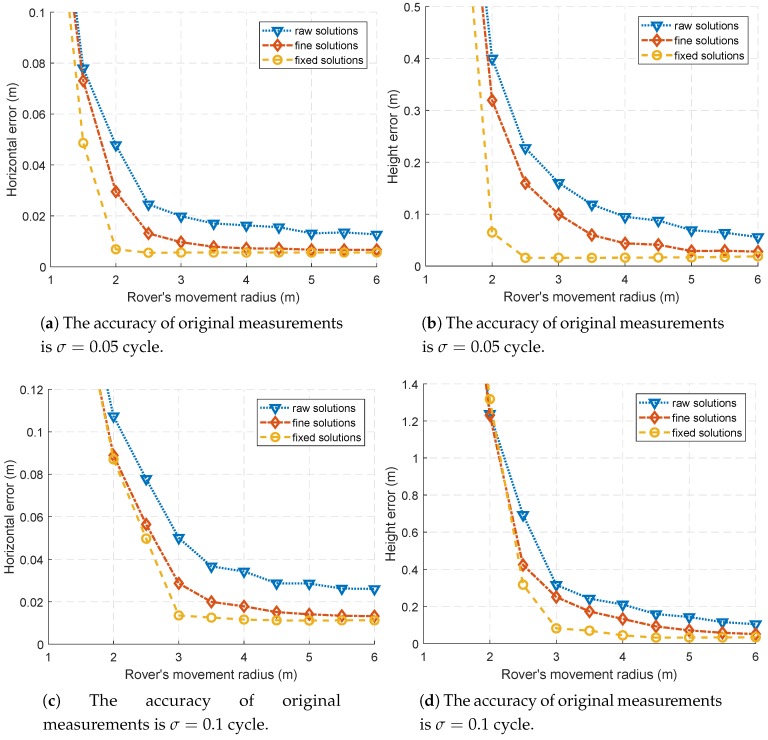
The rover’s movement radius *R* is increased from 1 m to 6 m, and the results with R<2 m are rather bad and not conducive to display.

The results of horizontal and height error are shown in Figure 4. The height error is nearly 10 times larger than the horizontal positioning error, and this is because the vertical dilution of precision (VDOP) is larger than horizontal dilution of precision (HDOP).

It can be seen from Figure 4 that the fixed solutions do not always achieve better positioning accuracy than floating solutions, such as when R=2 m and σ=0.01 cycles.

This is caused by the poor AR success rate. As shown in Figure 2a,b, when the movement radius *R* is too small, the fixed solutions are not reliable.

As the movement radius increases, the AR success rate gradually increases, and the positioning accuracy using fixed solutions can reach centimeter level.

It should be pointed that, when the movement radius is large, such as R≥5 m, the positioning accuracy of floating point solutions is not bad. As shown in Figure 4, the positioning accuracy of fine floating solutions could also achieve centimeter level. For R≥5 m, the positioning accuracy of fine float solutions and fixed solutions is quite close.

As shown by Figure 3, when the movement radius is large, the fine floating solutions are of centimeter level accuracy, resulting in a fairly good positioning accuracy shown in Figure 4.

This shows that the fine floating solutions may have great value in practical applications. For example, if there are some unknown model deviations in actual applications, the ambiguities might be treated as floating-point solutions rather than integer solutions.

For another example, if the AR success rate is too low, it might still be possible to reliably fix a subset of ambiguities, just like the partial ambiguity resolution in GNSS applications. This might be the future work.

## 5. Experiment Results

In order to verify the performance of the algorithm in the actual environment, an experiment was carried out. The experiment involved six prototype pseudolites and one user terminal, which are designed in a similar way as described in [29]. Taking into account regulatory issues of radio frequencies, the carrier frequency is set to be 2465.43 MHz.

**Figure 5:**
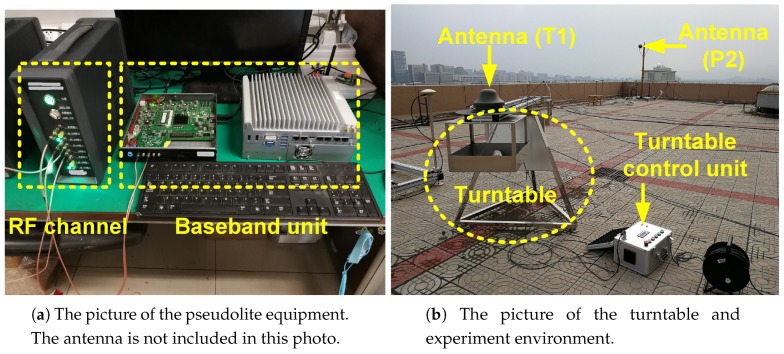
The picture of the prototype pseudolite system and the experiment environment.

The equipment composition of a pseudolite except for the antenna is shown in Figure 5a, including RF channel, baseband unit and management computer. Six pseudolites P1~P6 are stationary, and the coordinates of their antenna are measured by the total station and given in Table 1.

**Figure 6:**
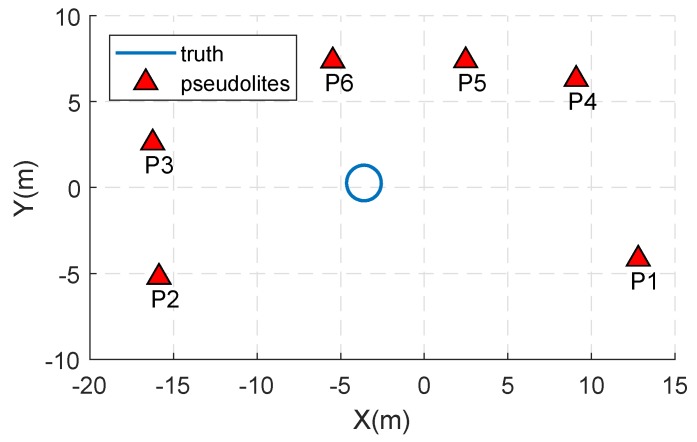
The horizontal diagram of the experiment configuration.

The antenna of the user terminal T1 is fixed on a turntable as shown in Figure 5b, and its position is to be determined. The center of the turntable is (−3.58, 0.27, 1.30), and its radius is 1.03 m. The configure diagram of the experiment is shown in Figure 6. During the whole movement, the HDOP ranges from 1.70 to 1.82 and the VDOP ranges form 5.78 to 7.02.

During the experiment, the rotational speed of the turntable is set as 0.314 rad/s, and the sampling rate is 10 Hz. The turntable rotates three turns in a counterclockwise direction and then three turns in a clockwise direction. The true ambiguity values are obtained by measurements in advance.

**Figure 7:**
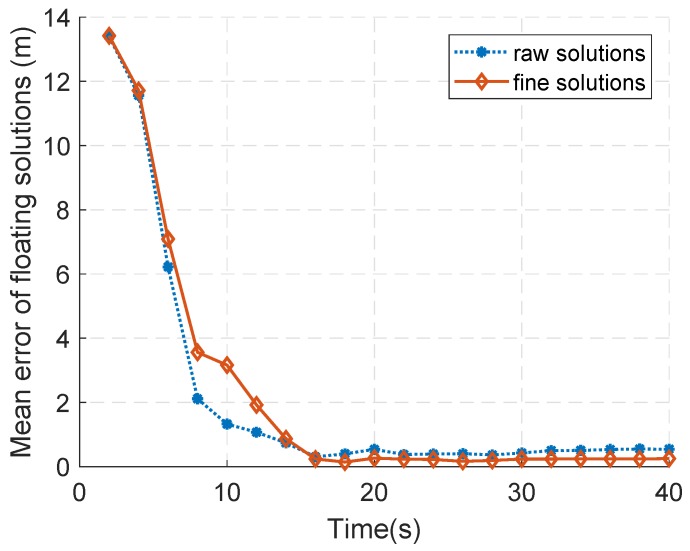
The horizontal diagram of the experiment configuration.

The mean error of the two floating solutions is shown in Figure 7. As can be seen, the error of floating solutions decreases rapidly with the rotation of antenna in the initial stage. When the rotation reaches one lap (that is, 20 s in Figure 7), the error of floating solutions does not rapidly decrease with the rotation as before. This can be because, while the antenna is still rotating, no significant geometric changes are introduced. In this case, the new observations fail to make a significant contribution to the precision of floating point solutions. During the second lap (20 s to 40 s in Figure 7), the errors of the raw solutions range from 36.7 cm to 55.1 cm, while those of the fine solutions range from 16.5 cm to 26.5 cm.

Another interesting phenomenon is that, during the initial three quarters of a turn (0∼15 s in Figure 7), the fine solutions seem to have a slightly larger error than the raw solutions. This is because the weighting information relies on the the rough position solutions obtained from the raw solutions. As a result, such information is not reliable enough when the motion range of the antenna is not large enough.

**Figure 8:**
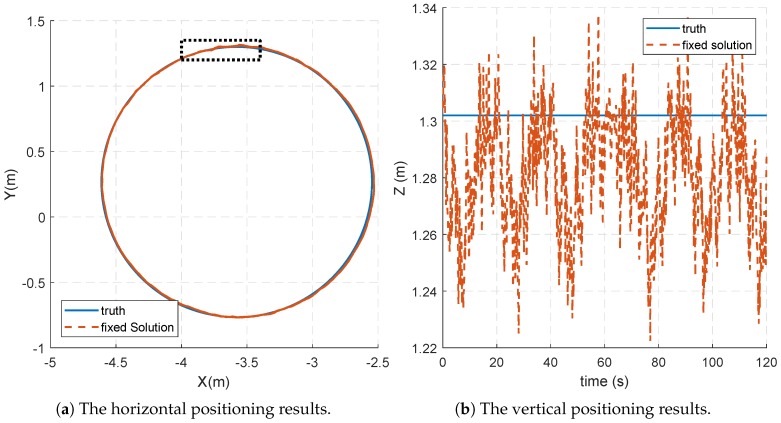
The comparison of the positioning results and the truth.

The proposed method correctly resolved ambiguities, and its practical value is proved by the positioning results. The positioning performance of the prototype pseudolite system is analyzed. Figure 8a shows the comparison between the horizontal positioning results and the true value of the trajectory. It can be seen that the positioning trajectory almost coincides with the true value. We compare the distance from the horizontal positioning results to the center with the known radius. In this sense, the root mean square error (RMSE) of horizontal results is about 0.83 cm.

**Figure 9:**
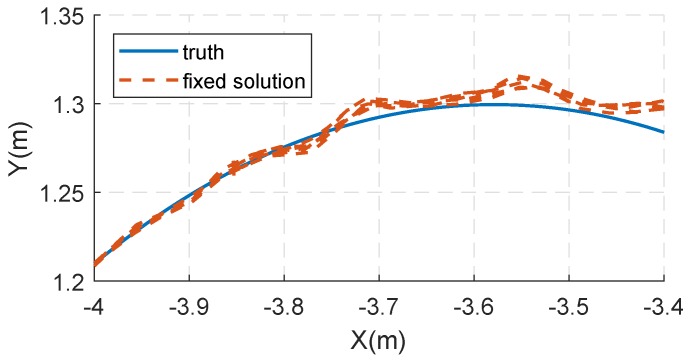
A partial enlargement of the horizontal positioning results.

Furthermore, a partially enlarged view of the horizontal trajectory is shown in Figure 9, and the corresponding part is indicated by the dashed box in Figure 8a. It can be seen that the positioning results of each turn have a high coincidence, and the positioning deviation is obviously related to the position.

This can be more clearly observed from the vertical positioning results in Figure 8b. It can be seen that the height errors approximately change in six cycles, which is directly related to the rotational position. The RMSE of the height results is about 2.92 cm compared to the true value.

The deviation of the positioning results is related to the rotational position, which is probably caused by multipath errors. In addition, the vertical errors are larger than the horizontal errors, and one of the reasons is that the VDOP is worse than HDOP. Another reason might be that the turntable is not strictly horizontal in the system coordinate, which will contribute to periodic deviations.

In summary, the experimental results show that the proposed algorithm can successfully solve the ambiguity, and the designed positioning system can achieve a centimeter-level positioning accuracy.

## 6. Conclusions and Future Work

A new OTF-AR method for ground based positioning systems using two-way ranging is proposed in this paper. The proposed method solely relies on carrier phase measurements, and applies to cases where no sufficiently accurate measurements are provided in advance, which is an important advantage.

One of the most important contributions is the concept of DDS observation discussed in this paper. Based on DDS observations, a linear model is established without an approximate linearization, which might cause significant nonlinear errors. It could provide a new way to utilize the geometric diversity for resolving ambiguities.

The proposed method is validated by numerical simulations and a real experiment. The results show that geometric changes have the most significant impact on AR performance. In the real experiment, the proposed algorithm has been successfully applied to our prototype pseudolite system, showing its application value.

Our future work will be directed towards extending the proposed method to general ground positioning systems without two-way measurements, where the rover no longer requires a transmitter. On the other hand, in practical application, gross error must be given careful consideration, although it is not the main focus of this paper and the current experiment is conducted under relatively good conditions. Multipath effect could be very serious in ground-based positioning. Interferences and occlusion should also be considered. The related study in GNSS can provide rich references, mainly including various statistical methods. In addition to efforts on the algorithm, the flexible design of pseudolites could provide various ideas for solving these problems.

Another meaningful topic is to analyze the performance bounds for the proposed method. Its main difficulty is that the variance matrix, which plays a key role in the LAMBDA algorithm, is estimated based on the floating point solutions.

## Figures and Tables

**Table 1:** The coordinates of six fixed pseudolites (unit: m).

	P1	P2	P3	P4	P5	P6
X	12.80	−15.87	−16.25	9.09	2.48	−5.47
Y	−4.17	−5.22	2.60	6.30	7.48	7.37
Z	3.24	3.27	3.20	3.28	4.58	4.83
